# Randomised controlled trial of intermittent vs continuous energy restriction during chemotherapy for early breast cancer

**DOI:** 10.1038/s41416-021-01650-0

**Published:** 2021-12-15

**Authors:** Michelle Harvie, Mary Pegington, Sacha J. Howell, Nigel Bundred, Phil Foden, Judith Adams, Lee Graves, Alastair Greystoke, Mark P. Mattson, Roy G. Cutler, Julie Williamson, Karen Livingstone, Debbie McMullen, Katharine Sellers, Cheryl Lombardelli, Grace Cooper, Sarah McDiarmid, Anthony Howell

**Affiliations:** 1 Prevent Breast Cancer Research Unit, The Nightingale Centre, Manchester University NHS Foundation Trust, Manchester, England; 2 Division of Cancer Sciences, The University of Manchester, Manchester, England; 3 Department of Medical Oncology, The Christie NHS Foundation Trust, Manchester, England; 4 Department of Medical Statistics, Manchester University NHS Foundation Trust, Manchester, England; 5 Centre for Imaging Sciences, University of Manchester, Manchester, England; 6 Research Institute for Sport and Exercise Sciences, Liverpool John Moores University, Liverpool, England; 7 Northern Institute for Cancer Research, Newcastle University, Newcastle upon Tyne, England; 8 Laboratory of Neurosciences, National Institute on Ageing Intramural Research Program, Baltimore, MD USA; 9 The Nightingale Breast Unit, Manchester University Hospital Foundation NHS Trust, Wythenshawe, Manchester, M23 9LT England

**Keywords:** Randomized controlled trials, Breast cancer, Nutrition, Weight management, Breast cancer

## Abstract

**Background:**

Excess adiposity at diagnosis and weight gain during chemotherapy is associated with tumour recurrence and chemotherapy toxicity. We assessed the efficacy of intermittent energy restriction (IER) vs continuous energy restriction (CER) for weight control and toxicity reduction during chemotherapy.

**Methods:**

One hundred and seventy-two women were randomised to follow IER or CER throughout adjuvant/neoadjuvant chemotherapy. Primary endpoints were weight and body fat change. Secondary endpoints included chemotherapy toxicity, cardiovascular risk markers, and correlative markers of metabolism, inflammation and oxidative stress.

**Results:**

Primary analyses showed non-significant reductions in weight (−1.1 (−2.4 to +0.2) kg, *p* = 0.11) and body fat (−1.0 (−2.1 to +0.1) kg, *p* = 0.086) in IER compared with CER. Predefined secondary analyses adjusted for body water showed significantly greater reductions in weight (−1.4 (−2.5 to −0.2) kg, *p* = 0.024) and body fat (−1.1 (−2.1 to −0.2) kg, *p* = 0.046) in IER compared with CER. Incidence of grade 3/4 toxicities were comparable overall (IER 31.0 vs CER 36.5%, *p* = 0.45) with a trend to fewer grade 3/4 toxicities with IER (18%) vs CER (31%) during cycles 4–6 of primarily taxane therapy (*p* = 0.063).

**Conclusions:**

IER is feasible during chemotherapy. The potential efficacy for weight control and reducing toxicity needs to be tested in future larger trials.

**Clinical trial registration:**

ISRCTN04156504.

## Introduction

Excess weight and adiposity at the time of breast cancer (BC) diagnosis and gains during treatment are linked to poorer outcomes [1]. Approximately 20,000 early BC patients receive chemotherapy each year in the United Kingdom [2]. Gains in weight and body fat and loss of lean body mass are common amongst such women [3]. These changes are associated with increased chemotherapy toxicity and thus dose reductions that may worsen outcomes [4, 5]. There are little data on weight control programmes during chemotherapy. These have typically involved continuous (daily) energy-restricted diets with limited success [6]. A recent review reported modest weight loss success in two out of five interventions during chemotherapy [7]. Likewise, our recent Breast-Activity and Healthy Eating after Diagnosis-1 (B-AHEAD-1) study showed that a supervised and home-based daily energy-restricted weight control programme controlled weight amongst patients receiving adjuvant radiotherapy and endocrine therapy, but not amongst women receiving chemotherapy. Thus, alternative approaches are required for patients receiving chemotherapy [8]. We have reported that intermittent energy and carbohydrate restriction (2 consecutive days/week; 650–1000 kcal, 50 g carbohydrate) is more effective for reducing body fat and improving insulin sensitivity than daily energy restriction amongst healthy women with overweight/obesity [9]. This intermittent approach may be easier for women to schedule around chemotherapy than a daily energy restriction. Furthermore, an intermittent energy restriction (IER) could help to manage chemotherapy toxicity and increase treatment efficacy. Short-term fasting and energy and protein restriction have been shown to reduce toxicity and increase the cytotoxicity of chemotherapy in some cell line and xenograft models [10, 11]. This is postulated to be mediated via reductions in glucose, insulin and insulin growth factor-1 (IGF-1) with associated down-regulation of Ras/MAPK and PI3K/Akt pathways and increased oxidative stress and autophagy [10, 11]. One small randomised controlled trial (RCT) (*n* = 13) testing a total fast for 24 h before and 24 h after chemotherapy reported higher erythrocyte and thrombocyte counts and lower levels of the DNA damage marker γ-H2AX in CD45+CD3− cells post chemotherapy, indicating potential reductions in bone marrow toxicity and DNA damage in peripheral blood mononuclear cells [12]. A recent randomised cross-over study of 50 women with breast and ovarian cancer reported that limiting to 350 kcal in the 36 h before and 24 h after chemotherapy administration reduced deteriorations in the quality of life and fatigue [13]. Whilst a recent RCT amongst 131 patients with HER2-negative stage II/III BC reported a higher odds of a radiological complete or partial response in patients using a low-energy, low-protein fasting-mimicking diet than women following their usual diet (odds ratio (OR) 3.168 *p* = 0.039) [14]. The B-AHEAD-2 study reported here aimed to compare the acceptability and effectiveness of IER compared with an isoenergetic continuous energy restriction (CER) group for weight control throughout the 4.5–6-month course of adjuvant/neoadjuvant chemotherapy. The comparator CER group provides an attention control group to allow us to ascertain the true effects of an IER vs CER as part of a supported weight control programme. Co-primary endpoints were changes in weight, body fat and fat-free mass (FFM). Secondary endpoints included chemotherapy toxicity.

## Materials and methods

### Study design

B-AHEAD-2 is a multicentre, randomised controlled two-arm (1:1) trial within ten breast units in the Greater Manchester Clinical Research Network, UK: Manchester University NHS Foundation Trust (MFT; the co-ordinating centre, The Christie, and hospitals in Stockport, Salford, Wigan, Oldham, North Manchester, Leighton, Macclesfield, Bolton and Tameside).

### Patient population

Participants were recruited prior to commencing adjuvant or neoadjuvant chemotherapy for stage 1–3 BC. Inclusion criteria included female sex, age ≥18 years, haemoglobin >110 g/L and body mass index (BMI) > 19 kg/m^2^ since the diet and physical activity (PA) plan could lead to weight loss amongst healthy weight individuals. Exclusion criteria included the presence of metastatic disease, chemotherapy for breast or other cancers within the last 2 years, physical or psychiatric conditions that could limit compliance to the diet and PA programmes and regular medication known to affect body composition such as daily corticosteroids. Women with diabetes were eligible unless receiving insulin or sulfonylureas due to the potential for hypoglycaemia on restricted days of IER. Women were invited to enter the study at either their first post-operative or first chemotherapy assessment appointment by clinicians, research or breast care nurses in the breast units.

### Randomisation and stratification

Randomisation to two groups (IER and CER) was undertaken by the trial administrator at MFT using a minimisation programme and was stratified by: adjuvant or neoadjuvant chemotherapy, BMI < 25 or ≥25 kg/m^2^ and pre-/peri- or post-menopausal.

### Study interventions

**Fig. 1:**
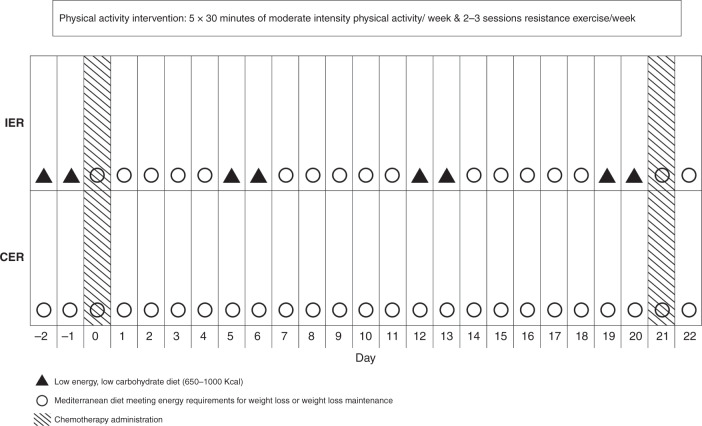
Description of the IER and CER interventions across one chemotherapy cycle

The isoenergetic IER and CER interventions are described in Fig. 1. Women were asked to commence their allocated diet (IER or CER) plus a PA programme prior to the first chemotherapy cycle and follow this throughout their prescribed chemotherapy course during the chemotherapy and non-chemotherapy weeks. Women in both groups with BMI ≥ 25 kg/m^2^ were recommended to achieve gradual weight loss of ≥5% (i.e. ≥3 kg reduction in body fat) by following a 25% energy restriction diet, whilst those with a BMI < 25 kg/m^2^ were recommended to maintain their weight and limit gains in body fat to <1 kg by meeting their estimated energy requirements. Baseline energy requirements were predicted using Henry equations [15] multiplied by their reported activity level in metabolic equivalents [16].

### Intermittent energy restriction

This involved two consecutive low-energy, low-carbohydrate days, which provided 650–1000 kcal, 50 g carbohydrate, 50–70 g protein, 30–40 g fat and include low-fat, high-protein foods (i.e. meat, fish, eggs, tofu, textured vegetable protein), monounsaturated (MUFA) and polyunsaturated (PUFA) fat, low-fat dairy foods, 5 portions of vegetables and 1 portion of fruit, and at least 2 L of low-energy fluids daily. A Mediterranean diet was recommended for the remaining 5 days of the week, which was tailored so that overall weekly intake matched their energy requirements for weight loss or maintenance. The Mediterranean diet has been described previously [9]. This provides 30% energy from fat (15% MUFA, 8% PUFA, 7% saturated), 25% energy from protein and 45% from low glycaemic load carbohydrate, and includes 5 portions of vegetables and 2 portions of fruit per day, and limits alcohol to <10 U/week.

Low-energy days were scheduled on the 2 days immediately prior to the day of chemotherapy infusion during chemotherapy weeks. Women were advised to return to the Mediterranean diet on the day they received chemotherapy.

### Continuous energy restriction

Women in this group were asked to follow the Mediterranean diet as above every day of the week tailored to their energy requirements for weight loss or maintenance.

### Dietary advice and support for both groups

Food and drink for IER and CER were self-selected and not provided by the study team. Neither group were asked to count calories. They were provided food lists and provided personalised advice on numbers of specified portions of carbohydrate, protein, fats, dairy, fruit and vegetables to achieve their energy prescription and associated menus and recipes. The advice was also given to manage any chemotherapy-related side effects such as nausea, mucositis, constipation and diarrhoea, as well as food hygiene advice.

Both groups received individualised diet and PA advice with an initial face-to-face consultation with a dietitian and cancer PA specialist at MFT. Weight, diet and PA goals were reinforced remotely with fortnightly phone calls from their designated trial dietitian to check adherence and address individual concerns. Each participant was posted an individualised summary of key motivational, behavioural, diet and PA issues after each call. They also received 12 standard fortnightly mailings that covered aspects of their allocated diet, weight management, PA and chemotherapy (Supplementary Table 1).

Both programmes used established behaviour change techniques including goal setting, self-monitoring of weight and waist, diet and PA, seeking personal support, getting back on track, vigilance for portion sizes and forming habits [17]. They both included the following safety protocols: (A) oncology teams were informed of patients with weight loss of ≥10% such that chemotherapy dose reduction could be considered if the toxicity of grade 2 or higher was experienced, (B) study dietitians advised increased energy intake if BMI reduced below 19 kg/m^2^ and (C) patients were advised to refrain from a moderate or vigorous activity if haemoglobin levels reduced below 90 g/L

### PA advice

Participants were advised to gradually increase the frequency and intensity of cardiovascular PA with the aim of undertaking 2.5 h (5 × 30 min) of moderate-intensity PA per week (at 60–80% maximum heart rate). Also, to undertake a progressive resistance PA programme according to published guidelines for patients receiving adjuvant chemotherapy [18]. PA advice was provided face to face by a cancer exercise specialist. The resistance exercise programme was demonstrated to patients as part of this consultation.

Cardiovascular PA advice was tailored according to participants’ current activity levels, preferences, abilities, co-morbidities and energy levels.

### Outcome measures

All outcome measures were measured before the start of chemotherapy (baseline) and 3 weeks after the final chemotherapy cycle (post chemotherapy) to avoid the acute effects of chemotherapy and the co-prescribed dexamethasone within the post-chemotherapy assessments. The primary outcome measures were changes in weight, body fat and FFM. Weight was assessed using a segmental multi-frequency body composition monitor (Tanita MC-180MA scale, Tanita Europe, Amsterdam, The Netherlands) and body fat and FFM were estimated from supine dual-energy x-ray absorptiometry (DXA) using Hologic A Discovery software (Hologic Inc., Marlborough, MA, USA) and corrected for unilateral artefacts, i.e. metallic implants and lymphoedema, as described previously [8]. Changes in total body water were estimated using the Tanita MC-180MA (at the time of the DXA scan) under standardised conditions, i.e. after a 12 h fast, avoidance of vigorous activity and alcohol in the previous 24 h and immediately after emptying the bladder.

Cardiovascular disease (CVD) risk factors (waist and hip circumference, DXA trunk fat, blood pressure, fasting insulin, glucose, homeostatic model assessment (HOMA), total low-density lipoprotein (LDL) and high-density lipoprotein (HDL) cholesterol, triglycerides) and appendicular lean mass were measured using standardised methods at MFT as described previously [8]. Exploratory metabolic (serum adiponectin and receptors for IGF-I, insulin and leptin), inflammatory (high-sensitivity C-reactive protein) and oxidative stress markers (advanced oxidation protein product [AOPP]: total protein ratio, uric acid, paraoxonase 1 [PON1]) were analysed at the National Institute on Aging Intramural Research Program (Baltimore, MD, USA). Levels of adiponectin in plasma and IGF-1 receptor, leptin receptor and insulin receptor (A and B) in serum were measured by immunoassays using commercially available kits according to the manufacturer’s protocols (Meso Scale Discovery Diagnostics, Gaithersburg, MD, USA). The following assays were also used: AOPP (Cell Biolabs, San Diego CA, USA, coefficient of variation [CV] 2.2%), total protein (Biuret assay, CV 2.31%) and uric acid (Randox Laboratories, Crumlin, UK, CV 2.01%); paraoxonase 1 (ZeptoMetrix Corporation, Buffalo, NY, USA, CV 2.71%).

Post-chemotherapy assessments in the IER group were undertaken at least 5 days after energy-restricted days to avoid any acute effects on weight or blood markers, which we have previously reported [19]. Biochemical data analysis included days of the menstrual cycle was relevant to adjust for variations across the menstrual cycle. All serum, plasma and whole blood samples were stored at −80 °C until completion of the study when analysis took place to reduce inter-batch variation.

Fitness was measured from distance walked in a 6-min treadmill walk test [20], forced vital capacity (FVC) and forced expiratory volume (FEV1) from an average of three spirometry measurements (Micro spirometer, Micromedical UK). Hand grip on the dominant side was assessed with an average of three measurements using a JAMAR Hand Dynamometer [21].

Quality of life was assessed with the Functional Assessment of Cancer Therapy (FACT) physical well-being (PWB), functional well-being (FWB), BC-specific (BCS), endocrine (ESS) and fatigue (FSS) sub-scales. These were reported as the trial outcome indicator (TOI) summary scores, e.g. TOI-BC = PWB + FWB + BCS; TOI endocrine symptoms (TOI-ES) PWB + FWB + ESS; TOI fatigue (TOI-F) = PWB + FWB + FSS [22].

### Diet and PA behaviours

Adherence to the diet and PA regimens was assessed from 7-day food diaries using Wisp version 3 (Tinuviel Software, Anglesey, Wales) and the International PA Questionnaire long version, presented as metabolic equivalent of task (MET) minutes. This represents the amount of energy expended carrying out PA as multiples of resting energy expenditure where walking equals 3.3 METS/min and moderate PA is 4 METS/min [23]. These were recorded at baseline and post chemotherapy in addition to 3 weeks during either chemotherapy cycle 3 or 4. Women in the IER group were asked to record completion of energy-restricted days each week throughout the study and whether these had been undertaken on the 2 days prior to chemotherapy administration.

### Chemotherapy toxicity and completion

Chemotherapy toxicity from the preceding cycle was recorded and graded by research staff in the breast units using CTCAE v4.0 [24]. We report the incidence of grade 3/4 toxicity across all cycles and during cycles 1–3 and 4–6. Chemotherapy relative dose intensity (RDI) was determined as a fraction of the original planned dose in mg/m^2^/week as well. We also collated the total number of nights of hospital stay throughout chemotherapy from a review of hospital records.

### Chemotherapy toxicity biomarkers

Serum cytokeratin 18 (CK18) and plasma FMS-like tyrosine kinase 3 ligand (FLT3 ligand) were measured to assess epithelial and bone marrow toxicity, respectively, in 26 IER and 20 CER patients. Samples were collected on days 1 and 8 of both cycles 1 and 6 to assess acute chemotherapy toxicity effects in these cycles. Samples were analysed at the Northern Institute for Cancer Research, Newcastle University. Samples were non-fasted, but women were asked to refrain from exercise for 4 h prior to sample collection [25].

### Exploratory BC outcomes

We report BC death, metastases and new primary BC for participants after a median of 70-month follow-up. In addition, we reported whether there was a complete pathological response for the subset of patients receiving neoadjuvant chemotherapy.

### Statistics and sample size

Allowing for 20% attrition, the sample size of 85 participants per group was chosen to provide 90% power to detect differences in the change in body fat during chemotherapy between the two groups of ≥2.0 kg (based on a simple *t* test with an SD of change in body fat in our B-AHEAD-1 control group of 3.5 kg). A detectable difference of 2.0 kg in body fat is achievable and clinically significant. Such changes in body fat are seen alongside a reduced weight of 2.7 kg (~3% weight loss), which has been linked to 24% reduced recurrence in the Women’s Intervention Nutrition Study [26]. We estimated that these numbers would allow us to have 80% power to detect a decrease in the prevalence of severe toxicity (grade 3/4) from 30% down to 10% and a difference in change in weight of >2.0 kg.

Laboratory staff undertaking blood assays were blinded to the participant’s study arm. It was not possible to blind other staff. Women who withdrew from the study consented to their chemotherapy toxicity, chemotherapy dose, post-chemotherapy weight and hospital admission data being used in the final analyses, which allowed complete case analysis.

### Statistical analysis

Statistics were undertaken with STATA 13 (Stata Corp, College Station, TX) using an intention-to-treat analysis. Multiple imputations were used to estimate missing values for the primary endpoints weight and body fat, and the secondary endpoints CVD risk markers and fitness using chained equations with variables selected for each model as reported in Supplementary Table 2. Fifty imputation datasets were used to ensure there was sufficient power and robustness in the results. Analysis of covariance models was used to compare changes in outcomes between the two groups, adjusting for baseline levels. Marginal means (95% confidence interval [CI]) for each diet group reflect a change from baseline.

We conducted two secondary predefined analyses. Since post-chemotherapy weights and DXA body composition assessments may be influenced by fluid retention [27], we analysed changes in weight and body fat and FFM using an ANCOVA model, which included post-chemotherapy total body water, which is the sum of both intracellular and extracellular water (estimated with bioelectrical impedance). We also compared changes in weight and body composition in the two groups stratified by BMI < 25 kg/m^2^ (35% of the cohort) or ≥25 kg/m^2^ (65% of the cohort).

Per-protocol analyses were used to report any differences between the diet groups for changes in quality of life, dietary intake (ANCOVAs adjusted for baseline values), PA, exploratory markers of metabolism, inflammation, oxidative stress and chemotherapy toxicity (Mann–Whitney). We also identified predictors of withdrawal from the study using multivariable logistic regression and a priori variables including an allocation to IER or CER demographic, treatment variables and age.

## Results

**Fig. 2:**
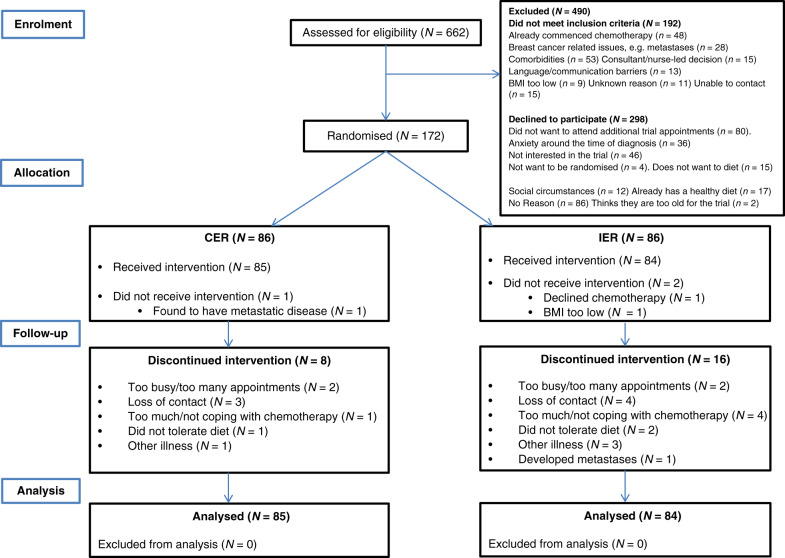
Consolidated Standards of Reporting Trials (CONSORT) flow diagram of patients recruited to the B-AHEAD-2 trial.

One hundred and seventy-two patients with early BC were recruited from the ten units between May 2013 and September 2014 representing 37% of eligible women (Fig. 2). Two patients of the IER group and one of the CER group were found ineligible and left the study prior to receiving the interventions. The remaining cohort included 158 women scheduled to receive adjuvant and chemotherapy and 11 receiving neoadjuvant chemotherapy. Patient characteristics are reported in Table 1.

**Table 1:** Baseline characteristics of patients randomised to the two groups.

	Intermittent (*n* = 84)	Continuous (*n* = 85)
Age at recruitment^a^	51.2 (31–71)	52.6 (24–77)
BMI at recruitment (kg/m^2^)^b^	28.0 (6.2)	28.2 (6.1)
BMI category^**c**^		
Healthy weight (19.0–24.9 kg/m^2^)	31 (36.9%)	29 (34.1%)
Overweight (25–29.9 kg/m^2^)	28 (33.3%)	31 (36.5%)
Obese (≥30 kg/m^2^)	25 (29.8%)	25 (29.4%)
Charlson Co-morbidity Index score^b^	2.2 (0.4)	2.1 (0.4)
Score ≤2^c^	71 (84.5%)	76 (89.4%)
Score >2^c^	13 (15.5%)	9 (10.6%)
Current smoker^c^	6 (7.1%)	3 (3.5%)
Menopausal status^c^		
Pre or peri/post	46 (54.8%)/38 (45.2%)	44 (51.8%)/41 (48.2%)
First-degree relative with BC^c^	20 (23.8%)	17 (20%)
Screen detected BC^c^	21 (25.0%)	20 (23.5%)
Days between final breast surgery and randomisation^d^	35 (9–72)	34 (18–174)
Tumour type and grade^c^		
Grade 3 carcinoma	54 (64.3%)	44 (51.8%)
Oestrogen receptor positive	59 (70.2%)	54 (63.5%)
HER2 receptor positive	19 (22.6%)	29 (34.1)
Triple negative	16 (19%)	16 (18.8%)
Surgery type^c^		
Mastectomy	35 (41.7%)	36 (42.4%)
Axillary node clearance	39 (46.4%)	39 (45.9%)
Chemotherapy regimen^c^		
Adjuvant/neoadjuvant	79/5 (94%/6%)	79/6 (94%/6%)
F(EC) and docetaxel/paclitaxel	81 (96.4%)	76 (89.4%)
Other, e.g. carboplatin or cyclophosphamide and docetaxel	3 (3.6%)	9 (10.6%)
Prophylactic G-CSF treatment^e^	84 (100%)	85 (100%)
Number of chemotherapy cycles	6 (4–10)	6 (4–8)
Ethnicity^c^		
White—all types	79 (94.0%)	78 (91.8%)
Other	5 (6.0%)	7 (8.2%)
Social circumstance^c^		
Married or co-habiting	64 (76.2%)	62 (72.9%)
Children living at home	45 (53.6%)	42 (49.4%)
Education^c^		
Educated to degree level or above	27 (32.1%)	27 (31.7%)
Employment		
Full- or part-time employment	58 (69.0%)	63 (74.1 %)
Retired or unemployed	26 (31.0%)	21 (24.7%)
*Index of multiple deprivation*^c,f^		
England quintiles		
1 (least deprived)	19 (22.6%)	17 (20.0%)
2	23 (27.4%)	23 (27.1%)
3	14 (16.7%)	16 (18.8%)
4	13 (15.5%)	13 (15.3%)
5 (most deprived)	15 (17.9%)	16 (18.8%)

Dexamethasone schedules: Docetaxal 8 mg b.d. for 3 days starting the day before chemotherapy.

Paclitaxel 8 mg i.v. pre-cycle 1 day 1 and 4 mg pre-cycle 1 day 8 only.

^a^Mean (range).

^b^Mean (SD).

^c^*N* (% of group).

^d^Median (range).

^e^Prophylactic G-CSF was not given with paclitaxel.

^f^Indices of Deprivation 2007 Layer Super Output Area Scores were identified from participant postcodes via Geoconvert10.

Sixteen of the IER group (19.0%) and eight of the CER group (9.4%) withdrew from the study (Fig. 2). Time to withdrawal was comparable between IER and CER (mean [SD] 11.6 [6.3] vs 12.4 [9.1] weeks, *p* = 0.82). Multivariable logistic regression identified that a lower risk of drop out was associated with older age (per year increase) (OR (95% CI) 0.93 (0.87–0.99), *p* = 0.022), first-degree relative with BC (0.13 (0.02–0.81), *p* = 0.029), education to degree level vs no degree (0.25 (0.07–0.93), *p* = 0.039), but not with allocation to DER vs IER (0.44 (0.15–1.2), *p* = 0.117).

### Participation in the IER and CER programmes

Women randomised to IER received mean (interquartile range) 87 (73–96)% of their expected calls and were posted 88 (78–96)% of their mailings, whilst women randomised to the CER group received 83 (66–87)% of their expected calls and were posted 93 (78–100)% of their mailings. No patients reduced BMI to <19 kg/m^2^ or had a haemoglobin of <90 g/L.

### Primary endpoints

#### Change in weight and body composition

Weight was measured in 169 women at baseline and 159 post chemotherapy (145 at MFT and 14 using post-chemotherapy weights in the breast units). DXA data were analysed from 150 participants at baseline (11 did not have a baseline scan and 8 had bilateral high-density artefacts) and 133 post chemotherapy, as 17 patients with baseline scans had withdrawn from the study.

ANCOVA analysis showed the difference in mean (95% CI) weight reduction with IER vs CER was −1.1 (−2.4 to +0.2) kg, *p* = 0.11, and body fat reduction with IER compared to CER was −1.0 (−2.1 to +0.1) kg, *p* = 0.086. In a predefined secondary analysis adjusted for body water, mean (95% CI) weight reduction in IER vs CER was −1.4 (−2.5 to −0.2) kg, *p* = 0.024 and body fat reduction was −1.1 (−2.1 to −0.2) kg, *p* = 0.046. Change in FFM was not significantly different between the groups. Both groups experienced comparable small increases in total body water (Table 2).

**Table 2:** Changes in weight, body composition and body measurements following chemotherapy.

		Baseline	Change 3 weeks post completion of chemotherapy^a^	IER vs CER^a^	Change 3 weeks post completion of chemotherapy adjusted for total body water^b^	IER vs CER^b^
All participants (*n* = 169)						
Weight (kg)	IER (*n* = 84)	73.8 (15.2)	−1.8		−1.9	
			(−2.7 to −0.8)		(−2.7 to −1.0)	
	CER (*n* = 85)	74.9 (17.2)	−0.7	−1	−0.5	−1.4
			(−1.6 + 0.2)	(−2.4 to +0.2)	(−1.3 to +0.3)	(−2.5 to −0.2)
				*p* = 0.11		*p* = 0.024
DXA subtotal fat (kg)	IER (*n* = 84)	28.9 (9.9)	−2.2		−2.3	
			(−3.0 to −1.4)		(−3.0 to −1.5)	
	CER (*n* = 85)	30.2 (11.2)	−1.3	−1	−1.2	−1.1
			(−2.0 to −0.5)	(−2.1 to 0.1)	(−1.9 to −0.5)	(−2.1 to −0.0)
				*p* = 0.086		*p* = 0.046
DXA FFM (kg)	IER (*n* = 84)	39.3 (6.1)	0.0		0.1	
			(−0.5 to 0.50)		(−0.4 to 0.5)	
	CER (*n* = 85)	39.1 (6.5)	0.5	−0.5	0.5	−0.4
			(0.1–1.0)	(−1.2 to 0.2)	(0.1–0.8)	(−1.0 to 0.1)
				*p* = 0.14		*p* = 0.14
Total body water (kg)	IER (*n* = 84)	32.9 (4.4)	1.8			
			(1.2–2.3)			
	CER (*n* = 85)	33.2 (4.7)	1.5	0.3		
			(1.0–2.0)	(−1.0 to 0.5)		
				*p* = 0.47		
Overweight participants (*n* = 131)						
Weight (kg)	IER (*n* = 66)	77.9 (14.5)	−2.0		−2.2	
			(−3.1 to −0.9)		(−3.2 to −1.1)	
	CER (*n* = 65)	79.9 (16.4)	−0.8	−1.2	−0.7	−1.5
			(−1.9 to 0.3)	(−2.8 to +0.4)	(−1.6 to 0.3)	(−2.9 to −0.1)
				*p* = 0.13		*p* = 0.038
DXA subtotal fat (kg)	IER (*n* = 66)	31.5 (9.6)	−2		−2.1	
			(−3.0 to −1.1)		(−3.0 to −1.2)	
	CER (*n* = 65)	33.4 (11.0)	−1.3	−0.7	−1.2	−0.9
			(−2.2 to −0.4)	(−2.0 to 0.5)	(−2.1 to −0.3)	(−2.1 to 0.3)
				*p* = 0.24		*p* = 0.15
DXA FFM (kg)	IER (*n* = 66)	40.8 (5.9)	−0.2		−0.2	
			(−0.8 to 0.4)		(−0.7 to 0.3)	
	CER (*n* = 65)	40.9 (6.1)	0.3	−0.5	0.3	−0.5
			(−0.2 to 0.9)	(−1.3 to 0.3)	(−0.2 to 0.8)	(−1.2 to 0.2)
				*p* = 0.19		*p* = 0.13
Normal weight participants (*n* = 38)						
Weight (kg)	IER (*n* = 18)	58.6 (5.0)	−1.2		−1.2	
			(−3.0 to 0.7)		(−2.8 to 0.4)	
	CER (*n* = 20)	58.5 (5.7)	−0.2	−0.9	−0.2	−1.1
			(−1.9 to 1.5)	(−3.5 to 1.6)	(−1.7 to 1.3)	(−3.3 to 1.1)
				*p* = 0.46		*p* = 0.33
DXA subtotal fat (kg)	IER (*n* = 18)	19.6	−2.5		−2.5	
		−3	(−4.2 to −0.90)		(−4.2 to −0.90)	
	CER (*n* = 20)	20	−1.3	−1.2	−1.3	−1.2
		−4.1	(−2.9 to 0.2)	(−3.5 to 1.1) *p* = 0.29	(−2.8 to 0.2)	(−3.5 to 1.0)
						*p* = 0.28
DXA FFM (kg)	IER (*n* = 18)	33.8	0.9		1.0	
		−3.5	(0.1–1.8)		(0.5–1.6)	
	CER (*n* = 20)	3.3	1.1	−0.2	1.0	0
		−3.6	(0.4–1.9)	(−1.3 to 0.9)	(0.5–1.6)	(−0.7 to 0.7)
				*p* = 0.70		*p* = 0.96

*FFM* fat-free mass.

^a^ANCOVA, mean (95% CI) using multiple imputation.

^b^ANCOVA, mean (95% CI) using multiple imputation adjusted for post-chemotherapy body water assessed with bioelectrical impedance.

Using the definition of weight maintenance as <3% weight change [28], there was a significant difference (*p* = 0.040) in the proportion of the IER and CER groups who lost weight (≥3% weight loss); 49.2% IER and 32.7% CER, maintained weight (<3% weight change) IER 31.2% and CER 49.6%, or gained weight (≥3% weight gain) IER 19.5% and CER 17.6%. Sensitivity analysis with complete cases produced results consistent with the imputed model (*p* = 0.036).

### Secondary endpoints

#### Chemotherapy toxicity and RDI

Grade 3/4 toxicities were reported in 31% of the IER and 36.5% of the CER groups across cycles 1–6 (*p* = 0.45), and respectively, in 21.4% vs 15.3% during cycles 1–3 (*p* = 0.30) and 17.9% vs 30% in cycles 4–6 (*p* = 0.063) (Table 3). A secondary analysis including patients receiving taxane therapy during cycles 4–6 (IER 81 CER 76) reported grade 3/4 toxicity in 14/82 (17.3%) IER and 24/77 (31.2% CER) (*p* = 0.037). Eighty IER (95%) and 80 CER (95%) patients achieved an RDI of ≥85%. Seven of the IER and one of the CER groups had a weight loss of ≥10%, but none had chemotherapy dose reductions as a result.

**Table 3:** Occurrence of grade 3 and 4 toxicities and hospital admissions.

	IER (*n* = 84)	CER (*n* = 85)	*P* value between groups
*Number of women with grade 3 and 4 toxicity*^a^			
*N* (% of women)^a^			
Cycles 1–6	26 (31.0)	31 (36.5)	0.45
Cycles 1–3	18 (21.4)	13 (15.3)	0.3
Cycles 4–6	15 (17.9)	26 (30.5)	0.063
*Number of grade 3 and 4 toxicity events*^b^			
Overall (cycles 1–6)			
Gastrointestinal symptoms^c^	16	15	
Febrile neutropenia/infection/low WCC	15	16	
Peripheral neuropathy	2	4	
Myalgia/arthralgia	3	8	
Other	1	5	
Cycles 1–3			
Gastrointestinal symptoms	12	8	
Febrile neutropenia/infection/low WCC	9	4	
Peripheral neuropathy	0	0	
Myalgia/arthralgia	0	1	
Other	0	4	
Cycles 4–6			
GI symptoms	6	9	
Febrile neutropenia/infection/low WCC	7	13	
Peripheral neuropathy	2	4	
Myalgia/arthralgia	3	7	
Other	1	2	
% of women with hospital admissions			
Total (cycles 1–6)	36.9	34.1	0.71
Cycles 1–3	25.0	17.6	0.24
Cycles 4–6	16.7	22.4	0.35

*WCC* white cell count.

^a^CTCAE-4. The number of women who experienced a grade 3/4 toxicity during the course of chemotherapy.

^b^The total number of women experiencing at least one grade 3/4 toxicity for each of the common toxicities. Patients could have a maximum of one score for each specific toxicity.

^c^Gastrointestinal symptoms include mucositis, nausea, vomiting, diarrhoea and constipation.

#### Cardiovascular disease risk markers and fitness tests

There were comparable reductions in the waist, hip, DXA trunk fat and increased appendicular lean mass in the IER and CER groups. Neither group experienced changes in HOMA or total and LDL cholesterol, whilst both experienced a reduction in HDL cholesterol. Serum triglycerides increased in the CER group and were maintained in IER. Mean (95% CI) change in triglyceride with IER vs CER was −0.193 (−0.388 to +0.002) (*p* = 0.053). Both groups reduced systolic blood pressure and FVC and maintained FEV1 and hand-grip strength. Change in distance walked in 6 min was not significantly different between the groups (Supplementary Table 3). Sensitivity analyses with complete cases produced results consistent with the imputed models (data not shown).

#### Quality of life

Both groups experienced comparable reductions in FACT-TOI-ES, TOI-F and TOI-BC (Supplementary Table 3).

#### Changes in self-reported dietary intake and PA

Complete analysis shows the IER group completed mean (95% CI) was 77 (71–84)% of the prescribed low-energy days throughout the course of chemotherapy. Intention-to-treat analysis assuming that women do not adhere to the diet after withdrawing from the study shows 67 (60–75)% days were completed. The majority of low-energy days undertaken (95%) were done as advised on the 2 days immediately prior to chemotherapy administration. The remaining 5% of days were undertaken on the day before and the day of chemotherapy administration.

Fifty-four of the IER (63%) and 66 of the CER (77%) group completed baseline and post-chemotherapy 7-day food diaries. Both groups had comparable reductions in intake of energy and fat, saturated fat and alcohol. The IER group had greater reductions in carbohydrate and maintained MUFA and protein compared to the CER group across the week (Supplementary Table 4). Within the IER group, mean (95% CI) energy and protein intake across the two scheduled low-calorie days each week were, respectively, 1185 (1084–1286) kcal and 80.2 (73.1–87.3) g/day, which were compared to 1462 (1367–1557) kcal and 73.3 (68.8–77.8) g/day for the five Mediterranean days. Mean (95% CI) energy intake across the 2 days immediately post-chemotherapy administration during cycle 3 or 4 was 1421 (1289–1554) kcal in the IER group and 1208 (1107–1309) kcal in the CER group. There were no changes in recorded PA in either group.

#### Markers of BC risk inflammation and oxidative stress

Both groups experienced reductions in adiponectin and IGF-IR and leptin receptor and increased insulin receptor levels. There were no changes in CRP, uric acid, PON1 or AOPP:total protein ratio in either group (Supplementary Table 5).

#### Chemotherapy toxicity biomarkers

Both groups experienced comparable increases in serum CK18 and plasma FLT3 ligand between days 1 and 8 within cycles 1 and 6. The IER group had a smaller reduction in the pre-chemotherapy level of FLT3, a biomarker of bone marrow toxicity, between cycles 1 and 6 (*p* = 0.033) (Supplementary Table 6).

#### Outcomes during extended follow-up

After a median of 70 months of follow-up, the IER group had ten BC events (five BC deaths, four distant metastases, one new primary BC) and one death from other causes. The CER group had ten BC events (five BC deaths, four distant metastases, one local recurrence) and one death from other causes. Amongst the 11 patients receiving neoadjuvant chemotherapy, all five of the IER patients had a complete response compared to two out of six of the CER group.

## Discussion

We report a large RCT of IER vs CER amongst women receiving chemotherapy for early BC and show that IER is feasible, but did not show significant reductions in weight and body fat compared to CER. These reductions were statistically significant after correction for body water, an important consideration since taxane chemotherapy, in particular, is known to increase fluid retention. There were no differences in grade 3/4 toxicity overall, but a trend for less grade 3/4 toxicity with IER vs CER during cycles 4–6 when patients received primarily taxane therapy (17.9 vs 30%). The findings build on previous work on strategies to control weight in this population by showing that IER is likely to be more effective than CER [8].

The known threshold for a clinically relevant weight difference is around 2.7 kg (3%). We have reported a difference of 1 kg between IER and CER. The greater reductions in weight and body fat with IER is likely to indicate this group achieved an overall greater energy restriction through greater dietary adherence compared to CER across the 5-month chemotherapy period. This is likely to be modest equating to a difference in intake of ~60 kcal/day, which is below the level of detection with dietary intake methodology. The higher dropout from IER was mainly associated with patient characteristics such as age and family history rather than dietary allocation. Previous studies in those receiving chemotherapy have reported equivalent attrition between IER and control groups [13]. Whilst studies in non-cancer settings have reported comparable attrition in those allocated to IER or CER [29].

The IER diet was prescribed to limit to 650–1000 kcal 2 days/week and approximately on average 1600 kcal (between 1400 and 1900 kcal) for the other 5 days of the week. The average intake on the restricted days was higher than prescribed, 1185 (1084–1286) kcal, whilst intakes were less than those prescribed during the non-restricted days, 1462 (1367–1557) kcal.

The resulting small difference in energy intake between IER and CER in the days before chemotherapy (1185 vs 1365 kcal) may limit the differences in toxicity reported between the groups. It is not known whether the effects of IER on chemotherapy toxicity would have been improved with better adherence to the prescribed low-energy days. Our results highlight the challenge of adherence to differential levels of energy restriction within a diet. Adherence to the restricted days could be enhanced by the provision of formula drinks [30]. However, a recent trial of a low-protein, low-carbohydrate formula, fasting-mimicking diet had low adherence (20% adherence during all eight cycles of chemotherapy). [14] The spontaneously reduced intake on non-restricted days was observed in previous trials [31].

The trend for reduced severe toxicity with IER during cycles 4–6 but not in the earlier cycles is of interest. This may be a chance finding or could be that higher toxicity rates in the second phase may increase the probability of seeing a difference. Alternatively, this could be a chemotherapy class-specific effect associated with taxane rather than anthracycline therapy. This class effect could be re-evaluated in a future confirmatory trial. De Groot et al. (*n* = 13) reported reduced haematological toxicity (erythrocyte and thrombocyte counts) with IER vs control amongst patients receiving combined taxane and anthracyline chemotherapy, but no improvements in grade 3/4 toxicity, which was reported in 6/7 patients following IER vs 3/6 controls [12]. A cross-over RCT tested a 60 h 350 kcal/day diet amongst patients with breast and ovarian cancer receiving taxane, platinum and anthracycline regimens. In this study, IER improved self-reported quality of life when administered in the first half of chemotherapy, but not when instigated during the second half [13]. IER may need to be undertaken all the way through chemotherapy to see the benefits in the later cycles.

This is the first study to assess the effects of IER on the toxicity biomarkers FLT3 and CK18. The apparent maintained FLT3 pre-cycle 6 with IER was not reflected in the self-reported clinical toxicity data and may be a chance finding in the subset. Alternatively, this could indicate better maintenance of bone marrow stem cells over the six cycles of chemotherapy. This is consistent with the CTCAE data showing reduced neutropenia with IER in the second half of chemotherapy, in addition to previously published preclinical [32] and clinical data [12].

This is one of the few studies to test energy-restricted weight control diets and to promote weight loss amongst women with overweight and obesity during chemotherapy. Most previous dietary studies have focussed on patients after they have completed adjuvant treatment. This misses an opportunity to offset deleterious changes in body composition and metabolism and to capitalise on a potential teachable moment around the time of diagnosis and during the initial cancer treatments [33]. The lack of data on the feasibility and safety of promoting intentional weight loss during chemotherapy means current guidelines recommend weight loss advice after completion of chemotherapy [34, 35].  Previous dietary intervention studies during chemotherapy have promoted weight loss amongst women with overweight and obesity with no obvious harms [36–38]. Notably, rates of toxicity and the RDI achieved in both our IER and CER groups are comparable to a similar population of women receiving these regimens as standard care in the absence of dietary interventions [39, 40].

Strengths of the study include the good uptake and inclusion of a representative sample of patients from ten breast units across a range of socioeconomic groups; the near-complete toxicity and chemotherapy dosage records allowing complete case analysis; limiting bias in the results; and the extensive correlative biomarker measurements. Limitations include the lack of a no-intervention control group to draw comparisons for changes in body weight and toxicity with the standard of care. However, our previous B-AHEAD-1 study reported that CER did not influence weight or body fat in a smaller number of women receiving adjuvant chemotherapy, and the primary research question of this study was, therefore, whether IER was more effective than CER, not control [8].

Gains in the extracellular fluid will influence post-chemotherapy estimates of weight and DXA FFM. Increased body water will explain the small gains in FFM. Similarly, the modest gains in appendicular lean mass are likely overestimates since DXA cannot distinguish accumulation of extracellular fluid from bone-free lean tissue [27]. Gains in body water were indicated by contemporaneous bioelectrical impedance measurements. Future studies could report phase angle and raw reactance and resistance measurements with bioelectrical impedance, which indicate cell membrane integrity and intra- and extracellular fluid compartments and have been linked to performance status and prognosis amongst patients with cancer [41, 42].

Anthracyline and taxane chemotherapies have been associated with modest gains in weight (1–3 kg) [43] and adverse changes in glucose and insulin sensitivity and other markers of the metabolic syndrome [44] in patients with early BC. These adverse metabolic effects are secondary to gains in adiposity and the acute effects of dexamethasone on glucose metabolism [45]. Also seen are increased oxidative stress and decreased antioxidant enzyme activity [46] evidenced by increased AOPP [47] and systemic inflammatory response. Our two test diets mainly attenuated weight gain and these adverse metabolic effects. Insulin and lipid markers were maintained in both groups. However, reductions in adiponectin and increased plasma insulin receptor post chemotherapy indicate reduced insulin sensitivity 3 weeks post chemotherapy, which is likely to be secondary to the acute effects of dexamethasone [45].

The IER group had greater numerical reductions in blood pressure and better post-chemotherapy scores for fatigue (FACT-TOI-F) and distance walked in the 6-min walk test than the CER group. The lack of statistical significance for these endpoints should not be interpreted as evidence of no difference between the two interventions since the current sample size had low statistical power (20–45%) for these endpoints.

The optimal timing of IER around chemotherapy is not known. Dorff et al. have reported a tendency for less toxicity with longer fasting periods, i.e. when total fasting was undertaken for 48 h before and 72 h (48 h before and 24 h after chemotherapy) compared to only 24 h before chemotherapy [48]. Our regimen involved two low-energy/low-carbohydrate days prior to chemotherapy infusion. We deliberately avoided the days after chemotherapy administration when adherence could be compromised by steroid-induced hyperphagia or any acute emetic effects of chemotherapy. The chemotherapy regimens utilised in the study have half-lives ranging from 15 min to 40 h. Refeeding during this period is likely to promote active DNA synthesis in healthy cells. This in turn increases their susceptibility to cytotoxic damage from chemotherapy [49]. Previous studies have reported increased colorectal and mammary tumorigenesis with exposure to carcinogens during refeeding of IER regimens [50]. Thus, extending energy restriction across this period could lead to greater reductions in toxicity. Importantly, our tested IER did not lead to hyperphagia on these days, which could worsen the situation.

Our timing of energy restriction prior to, but not after, chemotherapy administration avoids any theoretical reductions in cytotoxic effects on cancer cells alongside reductions in mitogens like insulin and IGF-1 and the available pool of proliferating cells as reported in preclinical studies with doxorubicin, fluorouracil, cyclophosphamide and docetaxel [51, 52]. However, our timing could miss any potential synergistic effects between energy restriction and chemotherapy. Preclinical studies suggest the DNA-damaging chemotherapy agent cisplatin is most effective amongst slowly proliferating cells [53]. Likewise, energy restriction has been shown to make cancer cells more susceptible to chemotherapy via activation of ATM/Chk2/p53 signalling pathway, oxidative stress and apoptosis [10]. The limited data for disease-free survival was comparable between the groups in this study. However, larger studies with longer-term follow-up are required to determine the impact of IER on recurrence and survival in patients receiving chemotherapy. The apparent greater complete response rate in the IER compared to the CER groups (5/5 vs 2/6) groups aligns with the recent findings of De Groot et al. [14]. However, the IER group included higher numbers of patients with an ER-Her2+ subtype than in the CER group (4/5 vs 0/6) who have a higher pCR rate [54]. The small numbers of neoadjuvant patients in the current trial limit the interpretation of these findings.

Previous dietary trials have tested short-term fasting [12] or low-energy, low-protein fasting-mimicking diets (350 kcal) [13]. Ongoing studies are testing a fasting-mimicking diet providing 750 kcal, 20 g protein, 100 g carbohydrate and 40 g fat [11]. Our low-energy, low-carbohydrate and maintained protein regimen (650–1000 kcal, 50 g carbohydrate, 70 g protein, 40 g fat) aimed to reduce levels of insulin [55] and maximise retention of lean body mass, which has a key role in chemotherapy toxicity [56]. We did not restrict protein as we did not specifically aim to lower serum IGF-1. Whilst a reduced IGF-1 level is important in the beneficial effects of fasting in preclinical models [57], insulin resistance is considered to be an equivalent or more important target than IGF-1 amongst patients with cancer [58, 59].

The phone intervention was effective for supporting dietary change, but did not increase PA. Notably reported baseline PA was maintained at 3 weeks post chemotherapy in both groups, perhaps indicating prevention of the normal 20% decreases alongside chemotherapy in these patients [60] Combined aerobic and resistance training during chemotherapy reduces short- and long-term side effects and some deconditioning effects of chemotherapy [61]. PA programmes can potentially increase the efficacy of chemotherapy through increased delivery, reduced intratumoural hypoxia, immune effects and increased apoptosis. Adherence to the PA intervention could be increased with partially supervised interventions, and possibly with the use of activity wearables self-monitoring and tailored feedback. Future studies of IER should include more intensive partially supervised PA interventions and test the benefits of IER vs PA vs a combined IER and PA intervention to test whether combined programmes produce synergistic effects on toxicity and outcomes of chemotherapy.

We demonstrate a low-energy, low-carbohydrate IER is feasible amongst women receiving chemotherapy for early BC with an indication of greater reductions in weight and body fat compared with CER. Future IER research should assess long-term BC and other health outcomes. These will be contingent on the effects of IER on chemotherapy efficacy and also maintained healthy weight and diet and PA behaviours beyond chemotherapy.

## Supplementary information

Reproducibility statement

Consort checklist

Supplementary table 1

Supplementary Table 2

Supplementary Table 3

Supplementary Table 4

Supplementary Table 5

Supplementary Table 6

## Data Availability

All datasets used and analysed during the current study are available from the corresponding author on reasonable request.
