# Supplementary material for: Randomised controlled trial of intermittent vs continuous energy restriction during chemotherapy for early breast cancer
Source: Br J Cancer. 2021 Dec 15;126(8):1157–67. doi: 10.1038/s41416-021-01650-0 (PMC9023522; doi:10.1038/s41416-021-01650-0)
Supplement: Supplementary file 6 — Supplementary Table 4 [file 41416_2021_1650_MOESM6_ESM.pdf]

Supplementary Table 4: Dietary intake and physical activity in the IER and CER groups for completers

	Baseline	Average during 3 weeks in chemotherapy cycle 3 or 4	P value[a]	3 weeks post end of chemotherapy	P value[b]
Energy (kcal)					
IER	1697 (1583-1813)	1398 (1302-1494)	0.86	1409 (1317-1500)	0.75
CER	1670 (1572-1770)	1365 (1283-1447)		1417 (1339-1494)	
Protein (g)					
IER	72.6 (68.2-77.1)	75.8 (70.9-80.8)	0.059	77.5 (72.4-82.6)	0.039
CER	72.0 (68.5-75.5)	68.3 (64.1-72.5)		71.2 (67.5-74.8)	
Carbohydrate (g)					
IER	199 (184-215)	145 (134-157)	0.04	151 (138-165)	0.034
CER	195 (182-208)	162 (151-172)		165 (155-176)	
Total fat (g)					
IER	67.9 (61.4-74.4)	59.0 (53.8-64.2)	0.10	55.4 (50.6-60.3)	0.42
CER	66.5 (61.4-71.7)	51.9 (48.0-55.8)		52.6 (48.6-56.7)	
Saturated fat (g)					
IER	24.7 (21.7-27.6)	20.1 (18.0-22.2)	0.45	18.8 (17.0-20.7)	0.96
CER	25.2 (22.7-27.6)	18.5 (16.8-20.2)		19.0 (17.2-20.8)	
Monounsaturated fat (g)					
IER	23.2 (20.9-25.5)	21.0 (19.1-22.8)	0.047	19.4 (17.6-21.2)	0.45
CER	22.1 (20.5-23.8)	17.7 (16.7-20.2)		18.3 (16.7-19.8)	
Polyunsaturated fat (g)					
IER	11.8 (10.7-12.9)	10.2 (9.2-11.2)	0.14	9.5 (8.5-10.6)	0.50
CER	10.5 (9.5-11.4)	8.7 (7.9-9.5)		8.7 (8.0-9.5)	
Alcohol (g)					
IER	0.3 (0.0-15.9) [d]	0.3 (0.0-5.1) [d]	0.10 [c]	0.0 (0.0-8.7) [d]	0.94 [c]
CER	0.7 (0.0-15.9)	0.3 (0.0-4.2)		1.1 (0.0-8.6)	
Fibre (g)					
IER	17.8 (16.2-19.5)	15.7 (14.4-17.0)	0.75	15.9 (14.2-17.5)	0.26
CER	16.3 (15.1-17.5)	16.2 (14.9-17.2)		16.2 (15.0-17.4)	
Physical activity (MET minutes/week)					

IER	2040 (972-3267) [d]	2123 (1305-3582) [d]	0.85 [c]	2131 (885-4070) [d]	0.34 [c]
CER	2577 (1316-4717)	2116 (1252-3896)		2468 (1605-4317)	

Mean (95% CI)

Dietary data: baseline and post chemotherapy IER n=54 CER n=66, baseline and mid chemotherapy IER n=48 CER n=61.

Physical activity baseline and post chemotherapy IER n=63 CER n=65), baseline and mid chemotherapy IER n=42 CER n=45.

a ANCOVA between IER and CER at mid chemotherapy cycle adjusted for baseline values

b ANCOVA between IER and CER at 3 weeks post chemotherapy adjusted for baseline values

c Mann Whitney of change in physical activity between baseline and mid chemotherapy cycle and baseline and post chemotherapy

d Median (25th and 75% centile)
